# A novel long noncoding RNA AC125257.1 facilitates colorectal cancer progression by targeting miR-133a-3p/CASC5 axis

**DOI:** 10.1515/med-2023-0631

**Published:** 2023-03-27

**Authors:** Chuanwen Liao, Zihan Zheng, Junye Liu, Jian Li, Rui Li, Shuqin Hu

**Affiliations:** Department of Gastrointestinal Surgery, Jiangxi Provincial People’s Hospital (The First Affiliated Hospital of Nanchang Medical College), Nanchang, Jiangxi, 330006, China; Department of Organ Procurement Organization, Jiangxi Provincial People’s Hospital (The First Affiliated Hospital of Nanchang Medical College), No. 152 Aiguo Road, Nanchang, Jiangxi, 330006, China

**Keywords:** CRC, AC125257.1, miR-133a-3p, CASC5, cell signaling

## Abstract

Colorectal cancer (CRC) is a common malignant gastrointestinal tumor. Long noncoding RNAs (lncRNAs) are revealed to be critically involved in CRC progression, providing new direction for exploring the pathogenesis of CRC. This study aimed to explore the biological functions and regulatory mechanisms of lncRNA AC125257.1 in CRC. Western blotting and reverse-transcription quantitative polymerase chain reaction were used for the measurement of gene expression. Cell counting kit-8 assay and flow cytometry analysis were used to explore the effects of AC125257.1 on CRC cell viability and apoptosis. RNA pull-down and immunoprecipitation assays were performed for validating the binding between AC125257.1 and its potential downstream microRNA. Results showed that lncRNA AC125257.1 expression was upregulated in CRC cells and tumor tissues. AC125257.1 enhanced cell viability and suppressed apoptosis of CRC cells. Moreover, the knockdown of AC125257.1 suppressed CRC progression *in vitro* and inhibited tumor growth *in vivo*. miR-133a-3p was revealed to bind with AC125257.1 in CRC cells. CASC5 was proved to be targeted by miR-133a-3p. Moreover, rescue assays indicated that the knockdown of AC125257.1 suppressed the pathogenic overexpression of CASC5. To conclude, AC125257.1 aggravates CRC development via miR-873-5p/CASC5 axis. Our findings might suggest a novel perspective that AC125257.1 may become the target for CRC treatment.

## 1 Introduction

Colorectal cancer (CRC) is known for high fatality rate as commonly diagnosed around the world [1]. Rectum adenocarcinoma (READ) and colon adenocarcinoma (COAD) are two CRC classifications with different anatomical locations and similar molecular mechanisms [2,3]. More than 90% cases of CRC are adenocarcinoma developed from colon and rectum glandular epithelial cells [4]. Although huge breakthroughs have been made on the pathogenesis and treatment of CRC, there still lack efficacious way to prevent and treat CRC. Hence, it is necessary to explore new targets for CRC therapy.

Long noncoding RNAs (lncRNAs) are reported to affect the cancer phenotypes by controlling cellular processes, such as apoptosis, proliferation, migration, and invasion [5]. With over 200 nucleotides in length, lncRNAs regulate expression via multiple ways, including microRNA (miRNA) sponge, epigenetic modifications, and mRNA stabilization [6]. In the competitive endogenous RNA (ceRNA) network, lncRNAs act as miRNA sponges competitively binding to miRNAs to inhibit the suppressive effects of miRNAs on the endogenous targets [7]. For example, lncRNA CASC21 promotes CRC cell proliferation, migration, and invasion and inhibits cell apoptosis by regulating the miR-7-5p/YAP1 axis [8]. lncRNA SNHG10 silencing suppressed the malignant behaviors of CRC cells by interacting with miR-3690 [9]. Recently, the lncRNA AC125257.1 was found to be autophagy related and contributed to poor prognosis in CRC [10]. The bioinformatics analysis also reveals that AC125257.1 is upregulated in CRC tissues compared with normal tissues. Nevertheless, the function and regulatory mechanism of AC125257.1 in CRC progression are largely unclear.

miRNAs are short noncoding transcripts (∼22 nucleotides) critically involved in the development of various diseases, including cancer [11]. Studies have suggested the role of miR-133a-3p as prognostic biomarker in sudden cardiac death, and miR-133a-3p is implicated in the ischemic myocardial injury [12,13]. lncRNA FGD5‑AS1 is indicated to attenuate LPS-caused sepsis by binding to miR-133a-3p to upregulate AQP1 [14]. Substantial evidence has also demonstrated that miR-133a-3p functions as a tumor suppressor in multiple cancers, such as esophageal squamous cell carcinoma, prostate cancer, and gastric cancer [15,16,17]. Moreover, previous studies have revealed that miR-133a-3p is downregulated in CRC tissues and its overexpression inhibits CRC cell proliferation [18]. It is also indicated as a biomarker of CRC [19]. Nevertheless, the findings of the previous study also revealed that SENP1 silencing partially rescued the suppressive effects induced by miR-133a-3p overexpression on CRC cell viability and proliferation, suggesting the existence of other potential mechanisms of miR-133a-3p in CRC, which requires further investigation [18].

Kinetochore scaffold 1 (CASC5, also named KNL1) is reported as an oncogene in lung adenocarcinoma and CRC [20,21]. It inhibits apoptosis and promotes the proliferation of CRC cells [21]. Bioinformatic analysis also reveals that CASC5 is significantly upregulated in the tissues of COAD and rectum adenocarcinoma (READ) patients and CRC patients with high CASC5 expression are predicted with poor overall survival.

In this study, we aimed to investigate the biological functions and regulatory mechanism of AC125257.1 in CRC. We hypothesized that AC125257.1 may affect CRC development via the miR-133a-3p/CASC5 axis. Based on gain- or loss-of-function assays, we explored the effects of AC125257.1 on CRC cell proliferation and apoptosis. The findings of our study may provide new insight into the pathogenesis of CRC.

## 2 Materials and methods

### 2.1 Patients and clinical specimens

CRC tissues (*n* = 36) and adjacent normal tissues (*n* = 36) were obtained from CRC patients receiving surgery at The First Affiliated Hospital of Nanchang Medical College. Adjacent normal tissues were collected 5 cm away from the cancerous tissues and confirmed by pathological examination. All participants signed the informed consent prior to the study. The information of patients and the correlation between AC125257.1 expression and clinicopathologic characteristics of CRC patients are presented in Table 1.

**Table 1:** Correlation between AC125257.1 expression and clinicopathologic characteristics of CRC patients

Characteristics	Number of patients (*n* = 36)	AC125257.1 expression		*P* value
		High (*n* = 22)	Low (*n* = 14)	
Gender				0.419
Female	15	8	7	
Male	21	14	7	
Age (years)				0.592
<60	11	6	5	
≥60	25	16	9	
Tumor location				0.796
Colon	20	10	10	
Rectum	16	12	14	
Tumor size (cm)				0.755
<5	14	9	5	
≥5	22	13	9	
TNM stage				<0.0001
I + II	16	4	12	
III	20	18	2	
Recurrence				0.003
No	17	6	11	
Yes	19	16	3	


**Ethics approval and consent to participate:** The experiments were approved by the Ethics Committee of The First Affiliated Hospital of Nanchang Medical College. The contents of this study are under full compliance with government policy and the Declaration of Helsinki.

### 2.2 Bioinformatics analysis

Interactive Analysis of Gene Expression Profiles, abbreviated as GEPIA, was used for RNA expression analysis (http://gepia.cancer-pku.cn/). Meanwhile, it provides customizable options, such as analysis of differential expression between tumor and normal patients, similar gene detection, and survival analysis [22]. The normal/tumor differential analysis and the prognostic value of AC125257.1 referred in our paper were all from GEPIA. Potential miRNAs targeting CASC5 (also named KNL1) were searched on the starBase database (https://starbase.sysu.edu.cn/) under the condition of CLIP-Data ≥ 5, Degradome-Data ≥ 0, pan-Cancer ≥ 10, and programNum ≥ 2. Only miR-133a-3p was screened out and subject to further analysis. lncRNAs possibly bound to miR-133a-3p was predicted on the starBase website under the condition of Degradome-Data ≥ 1.

### 2.3 Cell culture and transfection

Human colonic epithelial cells (HCoEpiCs, BFN608006386) and four other CRC cell lines from BLUEFBIO (Shanghai, China), including SW480 (BFN60800644), HCT116 (BFN607200665), Caco-2 (BFN60800651), and HCT-8 (BFN60800647), were cultured in flasks coated with Dulbecco’s modified eagle medium [10% (v/v) fetal bovine serum + 1% penicillin–streptomycin solution, Hyclone]. The culture environment was set at 37°C with 5% CO_2_. For cell transfection, sh-AC125257.1#1/2, sh-NC, miR-133a-3p mimics, NC mimics, and pcDNA3.1/CASC5, were designed by Ribobio Technology (Guangzhou, China). HCT-8 and SW480 were transfected with these vectors using Lipofectamine 2000 (Invitrogen, USA), complied with the product’s protocols.

### 2.4 Reverse-transcription quantitative polymerase chain reaction (RT-qPCR)

RNAs in CRC cells or tumor tissue were isolated using the RNeasy Mini Kit (Qiagen, Valencia, CA, USA), complied to the product’s protocols. RevertAid First Strand cDNA Synthesis Kit (Thermo Fisher Scientific) was used for reverse transcription. Applied Biosystems™ 7500 Fast Dx Real-Time PCR Instrument was used for qPCR process. The internal controls were U6 and GAPDH. The temperatures for denaturation, annealing, and elongation were set to 95, 60, and 72°C with 40 cycles. The quantitative calculation was performed using 2^−ΔΔCT^ method. Promega (Madison, WI, USA) synthesized all these primer sequences and the primers are shown in Table 2.

**Table 2:** Primers for RT-qPCR

Primer name	Primer sequence
GAPDH forward	5′-TCAAGATCATCAGCAATGCC-3′
GAPDH reverse	5′-CGATACCAAAGTTGTCATGGA-3′
U6 forward	5′-ATACAGAGAAAGTTAGCACGG-3′
U6 reverse	5′-GGAATGCTTCAAAGAGTTGTG-3′
AC125257.1 forward	5′-CGCTCTCCTTCAAGCTCCCG-3′
AC125257.1 reverse	5′-TGGAGAGGCTTCCTGCCCAT-3′
CASC5 forward	5′-ACTATAAAGGTATTCCAGACGG-3′
CASC5 reverse	5′-CAGAAAGCAATGTGTTCATCC-3′
miR-133a-3p forward	5′-TTTGGTCCCCTTCAACCAGCTG-3
miR-133a-3p reverse	5′-GCAGGGTCCGAGGTATTC-3′

### 2.5 RNA pull-down assay

A total of 1 × 10^7^ cells transfected with biotinylated miR-133a-3p mimic (bio-miR-133a-3p) or bio-NC were lysed. Magnetic RNA-Protein Pull-Down Kit was used for cultivation with magnetic beads. GAPDH was used as a negative control. CASC5 or AC125257.1 expression in precipitated RNA complex was measured by qPCR.

### 2.6 RNA immunoprecipitation (RIP)

The RIP assay was performed using EZ-Magna RIP™ RNA-Binding Protein Immunoprecipitation Kit (17-701; Millipore), complied with the product’s protocols. A total of 1 × 10^7^ HCT-8 and SW480 cells were lysed and centrifuged at 12,000*g* for 10 min. Supernatants were then co-incubated with magnetic beads containing anti-IgG (ab172730, 1:100; Abcam) or anti-Ago2 (ab186733, 1:50; Abcam) and then washed with 1,000 μL of RIP wash buffer. The isolated RNA was analyzed by PCR.

### 2.7 Cell counting kit-8 (CCK-8) assay

HCT-8 and SW480 were cultivated in 96-well plates (4 × 10^3^ cells/well). The corresponding cultivation time was 12, 24, and 48 h at 37°C. The cells were mixed with 10 μL of CCK-8 solution (Dojindo) at 37°C. After 2.5 h, the optical density (OD) value at 450 nm was detected using microplate reader.

### 2.8 Flow cytometry

The flow cytometry was performed using the Annexin V-FITC/propidium iodide (PI) Apoptosis Detection Kit (Yeasen, Shanghai, China). Cold phosphate-buffered saline (PBS) (Solarbio, Beijing, China) was used for washing. After washing twice with cold PBS buffer, the CRC cells were centrifuged at 4°C and 5 min later and made into 1 × 10^6^ cells/mL suspension with 100 L of 1× binding buffer. After 15 min of complete reaction with 10 μL of PI solution and 5 μL of Annexin V-FITC, the mixture was placed on ice for detection by flow cytometry (BD Biosciences, USA).

### 2.9 Western blot

The protein of CRC cells was extracted and quantitatively analyzed by the bicinchonininc acid method (Solarbio, Beijing, China). Sodium dodecyl sulfate-polyacrylamide gel electrophoresis (10%) was used for separation. After being transferred to polyvinylidene fluoride membrane (Micropore Company, USA) and sealed with 5% nonfat milk, the mixture was blocked with the tris buffered saline with Tween-20 buffer. After washing with Tween buffer thrice, primary antibodies were added for incubation at 4°C overnight. Then, it was mixed with goat anti-rabbit IgG secondary antibody (1:1,000, ab7090; Abcam, UK) at room temperature. Two hours later, enhanced chemiluminescence reagents (Bio-Rad, Hercules, CA, USA) were used for protein level detection. Primary antibodies were as follows: anti-GAPDH (1:2,500, ab9485), anti-CASC5 (1:1,000, ab70537)), anti-Bcl-2 (1:1,000, ab32124), anti-Bax (1:2,000, ab32503), anti-CDK4 (1:1,000, ab108357), anti-Cyclin A1 (1:1,000, ab270940), and anti-Cyclin D1 (1:1,000, ab16663).

### 2.10 Animal experiments

BALB/c male nude mice (5 weeks, 20 ± 2 g) were provided by the Vital River (Beijing, China). Mice were randomly divided into the sh-NC and sh-AC125257.1 groups. The cell suspension of HCT116 cells transfected with sh-NC or sh-AC125257.1 (1 × 10^6^ cells/mL) was subcutaneously injected into right axilla of each mouse. Tumor volume growth was monitored weekly by the following formula: volume = width^2^ × length/2. Mice were sacrificed at day 28, and tumors were obtained and weighed. The animal study has been approved by the Animal Care and Use Committee of The First Affiliated Hospital of Nanchang Medical College.

### 2.11 Statistics analysis

Data were analyzed using SPSS 19.0 and GraphPad 7.0 and expressed as the mean ± standard deviation. One-way analysis of variance was taken to compare means between three or more groups followed by Dunnett’s post hoc test. Student’s *t*-test was taken to compare means between two groups. Statistical significance was represented as *P* < 0.05.

## 3 Results

### 3.1 CASC5 was significantly expressed in CRC tissues and cells

**Figure 1:**
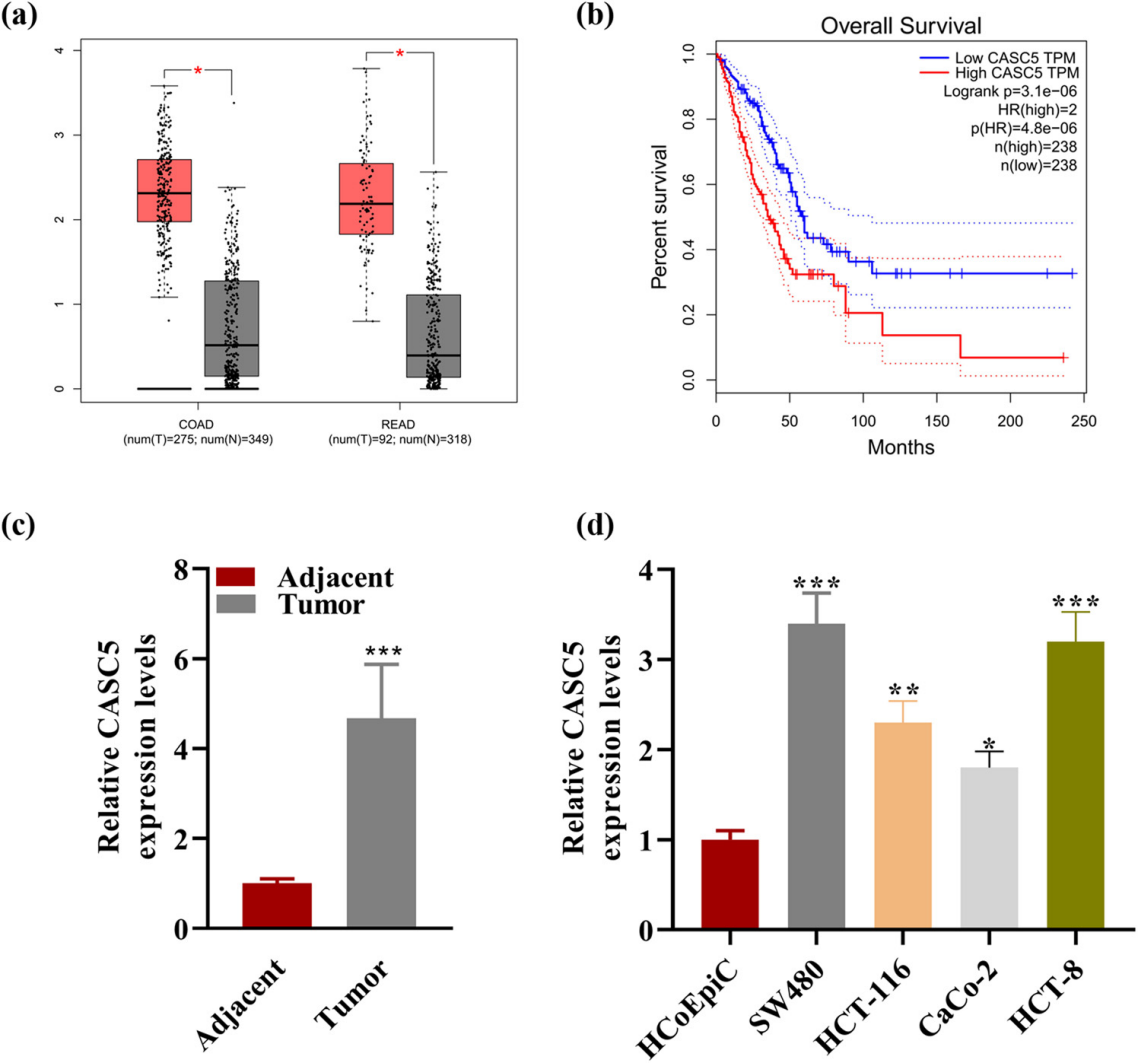
(a) The distribution of CASC5 in COAD (275 tumor samples and 349 normal samples) and rectum adenocarcinoma (READ, 92 tumor samples and 318 normal samples). Red column represented tumor samples, and gray column represented normal samples. (b) The overall survival curve of patients with low and high CASC5 TPM based on GEPIA database using Log-rank test. (c) Relative CASC5 expression in adjacent and tumor. (d) Relative CASC5 expression in HCoEpiC and four other CRC cell lines. *P < 0.05, **P < 0.01, ***P < 0.001.

From GEPIA database, high expression of CASC5 was found in CRC patients (either with COAD or with rectum adenocarcinoma) (Figure 1a). The overall survival curve showed that patients with high CASC5 level had worse prognosis than those with low level (Figure 1b). RT-qPCR results indicated that the expression of CASC5 was higher in tumor than in adjacent parts (Figure 1c) and higher in CRC cells than in HCoEpiCs (Figure 1d). These results revealed that CASC5 associated with CRC and has bad prognosis.

### 3.2 miR-133a-3p suppressed CASC5 expression through sponging

**Figure 2:**
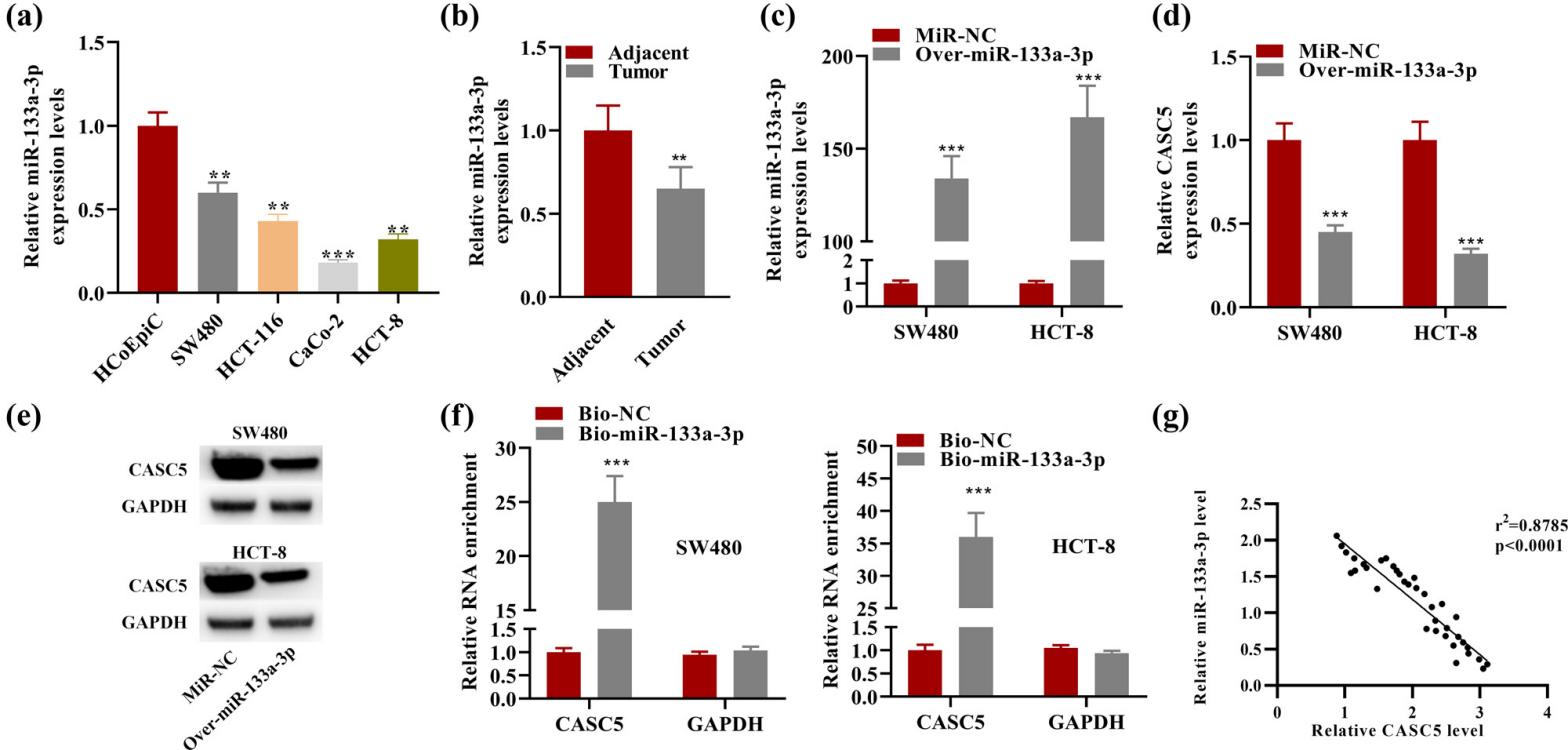
(a) Relative miR-133a-3p expression in HCoEpiC and four other CRC cell lines. (b) Relative expression of miR-133a-3p in adjacent and tumor. (c) Relative miR-133a-3p expression in SW480 and HCT-8 cell lines transfected with over-miR-133a-3p/miR-NC. (d) Relative CASC5 expression in SW480 and HCT-8 cell lines transfected with over-miR-133a-3p/miR-NC. (e) Western blot results of CASC5 in SW480 and HCT-8 cell lines transfected with over-miR-133a-3p/miR-NC. (f) RNA pull-down results were used to detect the enrichment of CASC5 and GAPDH (negative control) in the complex pulled down by bio-miR-133a-3p or bio-NC. (g) The expression correlation between miR-133a-3p and CASC5 in CRC patient tissues was explored using Pearson correlation analysis. **P < 0.01, ***P < 0.001.

miRNAs with potential binding site to CASC5 were predicted on the starBase database under the condition of CLIP-Data ≥ 5, Degradome-Data ≥ 0, pan-Cancer ≥ 10, and programNum ≥ 2. The results showed that only miR-133a-3p was screened out. RT-qPCR results showed lowly expressed miR-133a-3p in CRC cells (Figure 2a) and tumor samples (Figure 2b). In SW480 and HCT-8 with miR-133a-3p overexpression, the level of CASC5 expression was significantly downregulated (Figure 2c and d). Western blot results further confirmed this finding (Figure 2e). By using a bio-miR-133a-3p probe, RNA pull-down assay showed that CASC5 has high enrichment in the bio-miR-133a-3p group, demonstrating that CASC5 is targeted by miR-133a-3p (Figure 2f). Moreover, the expression of miR-133a-3p was demonstrated to be negatively correlated with CASC5 expression in CRC patient tissues (Figure 2g).

### 3.3 miR-133a-3p reduced CRC cell proliferation and promoted apoptosis while CASC5 partially reversed these effects

**Figure 3:**
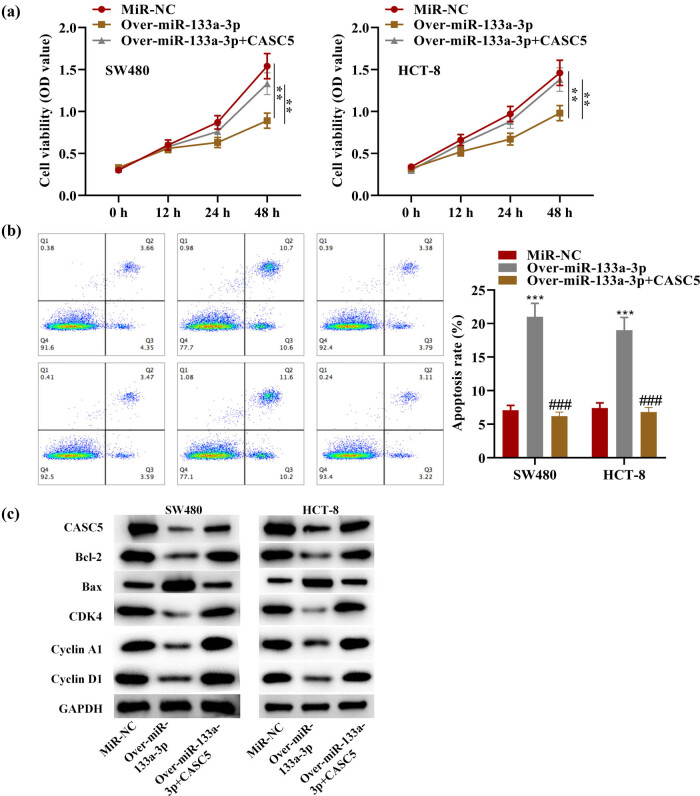
(a) OD value of 450 nm in SW480 and HCT-8 at 0, 12, 24, and 48 h. (b) Flow cytometry results and apoptosis rate of SW480- and HCT-8 transfected with miR-NC, over-miR-133a-3p, and over-miR-133a-3p + CASC5. (c) Western blot results of CASC5, Bcl-2, Bax, CDK4, Cyclin A1, Cyclin D1, and GAPDH in SW480 and HCT-8 transfected with miR-NC, over-miR-133a-3p, and over-miR-133a-3p + CASC5. **P < 0.01, ***P < 0.001 compared with the miR-NC group; ^###^ *P* < 0.001 compared with the over-miR-133a-3p group.

By CCK-8 assays, SW480 and HCT-8 transfected with over-miR-133a-3p showed suppressed proliferation, while those transfected with over-miR-133a-3p + CASC5 partially reserved this effect (Figure 3a). As is shown in flow cytometry results, the apoptosis rate of SW480 and HCT-8 transfected with over-miR-133a-3p was remarkably increased, while group transfected with over-miR-133a-3p + CASC5 showed reversed effect (Figure 3b). Further western blot analysis clarified the protein changes (Figure 3c). To be specific, CASC5, BCL2, and CDK4 levels were decreased by overexpressed miR-133a-3p, and BAX level was increased by miR-133a-3p, suggesting the promotive effects of miR-133a-3p on CRC cell apoptosis, further proving the results in Figure 3b. The levels of Cyclin A1 and Cyclin D1 were downregulated, suggesting that overexpressed miR-133a-3p suppressed CRC progression. These effects could all be restored partially by CASC5.

### 3.4 Knockdown of AC125257.1 promoted the expression of miR-133a-3p and suppressed that of CASC5

**Figure 4:**
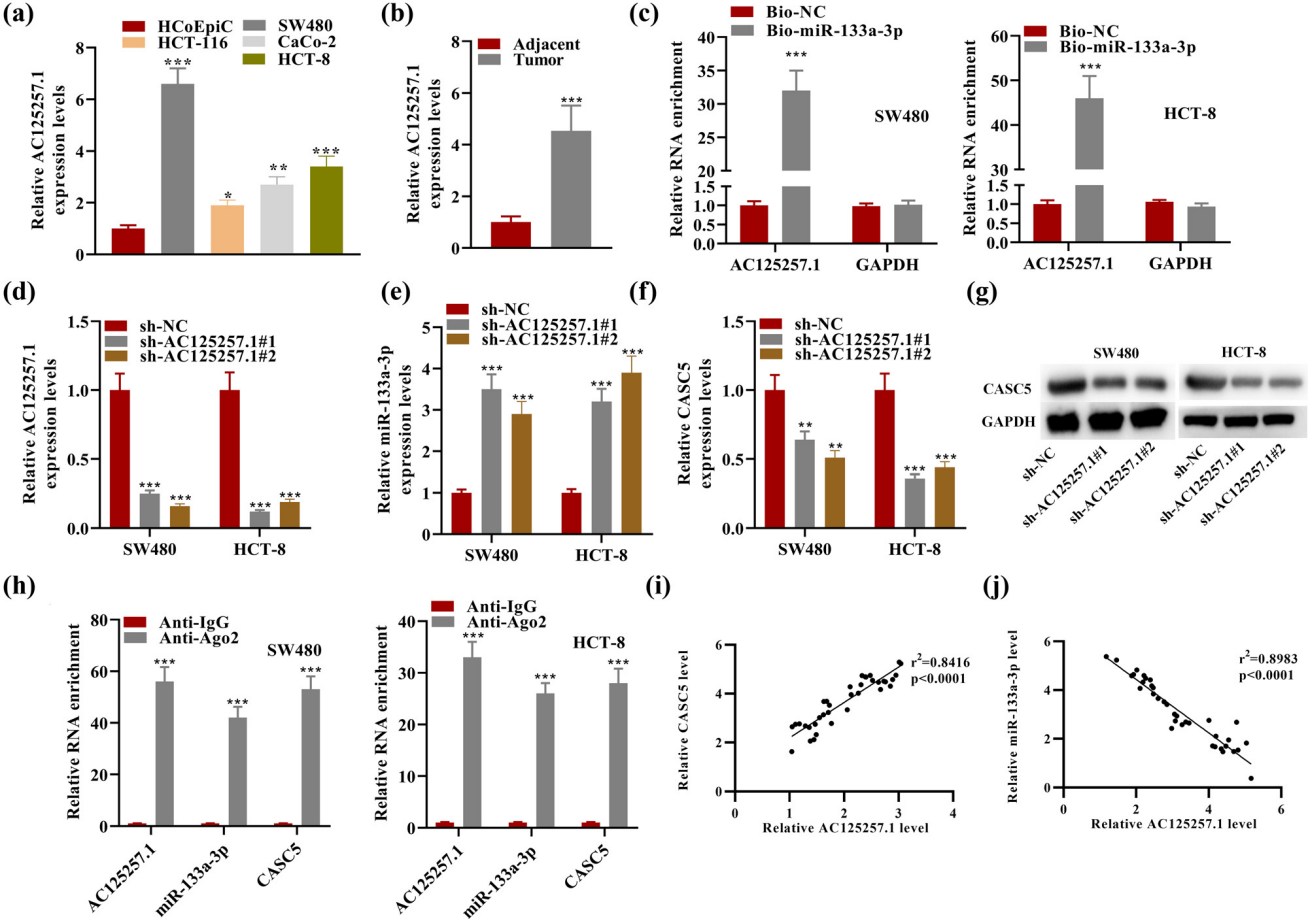
(a) Relative AC125257.1 expression in HCoEpiC and four other CRC cell lines. (b) Relative AC125257.1 expression in adjacent and tumor. (c) RNA pull-down results of AC125257.1 using bio-miR-133a-3p or bio-NC. (d) Relative AC125257.1 expression in SW480 and HCT-8 cell lines transfected with sh-NC/sh-AC125257.1. (e) Relative miR-133a-3p expression in SW480 and HCT-8 cell lines transfected with sh-NC/sh-AC125257.1. (f) Relative CASC5 expression in SW480 and HCT-8 cell lines transfected with sh-NC/sh-AC125257.1. (g) Western blot results of CASC5 and GAPDH in SW480 and HCT-8 cell lines transfected with sh-NC/sh-AC125257.1. (h) AC125257.1 and miR-133a-3p levels detected by RIP. (i) Pearson correlation analysis was conducted to examine the expression correlation between CASC5 and AC125257.1. (j) The expression correlation between AC125257.1 and miR-133a-3p in CRC patient tissues. *P < 0.05, **P < 0.01, ***P < 0.001.

lncRNAs possibly bound to miR-133a-3p was predicted on the starBase website under the condition of Degradome-Data ≥ 1. Only AC125257.1 was selected under this condition. AC125257.1 showed abundant expression in CRC cells and tumor by RT-qPCR (Figure 4a and b). RNA pull-down assay proved that AC125257.1 was remarkably enriched by the bio-miR-133a-3p probe, indicating that miR-133a-3p is the target miRNA of AC125257.1 (Figure 4c). As shown in Figure 4d–f, the knockdown of AC125257.1 significantly elevated miR-133a-3p expression and suppressed CASC5 expression, which is consistent with the finding in Figure 2c and d. Western blot results further confirmed this finding (Figure 4g). Furthermore, RIP assay validated that compared to the control group (anti-IgG), AC125257.1 and miR-133a-3p were noticeably enriched in microribonucleoproteins with Ago2, suggesting that AC125257.1 acted as the ceRNA for miR-133a-3p and formed an RNA-induced silencing complex in CRC cells (Figure 4h). The expression of CASC5 and AC125257.1 showed positive correlation in CRC patient tissues, while that of miR-133a-3p and AC125257.1 exhibited negative correlation according to Pearson correlation analysis (Figure 4i–g).

### 3.5 Knockdown of AC125257.1 reduced CRC cell proliferation and promoted apoptosis while CASC5 partially reversed these effects

**Figure 5:**
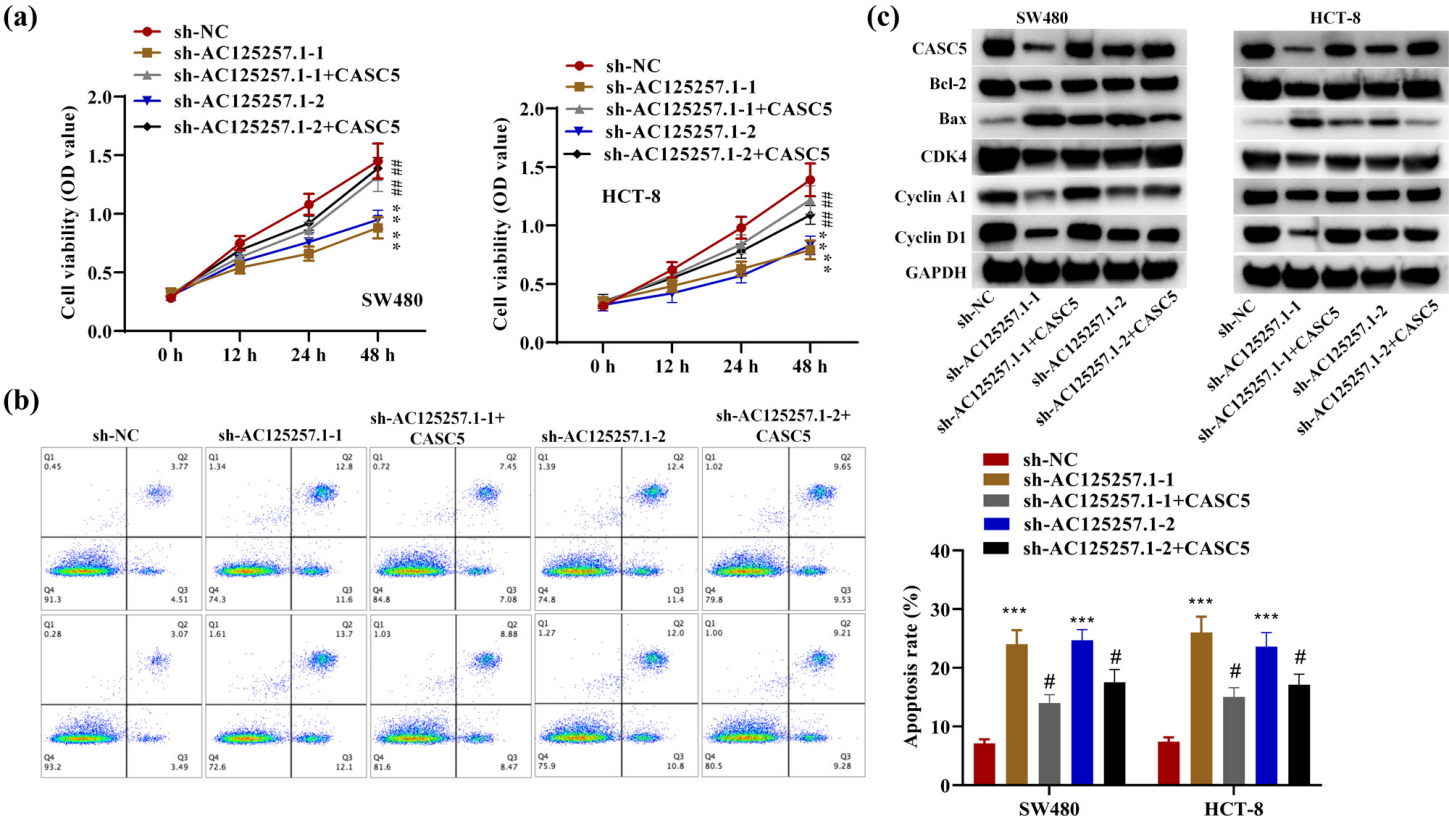
(a) OD value of 450 nm in SW480- and HCT-8-transfected with sh-NC, sh-AC125257.1, and sh-AC125257.1 + CACS5 at 0, 12, 24, and 48 h. (b) Flow cytometry results and apoptosis rate of SW480 and HCT-8 transfected with sh-NC, sh-AC125257.1, and sh-AC125257.1 + CACS5. (c) WB results of CASC5, Bcl-2, Bax, CDK4, Cyclin A1, Cyclin D1, and GAPDH in SW480- and HCT-8-transfected with sh-NC, sh-AC125257.1, and sh-AC125257.1 + CACS5. **P < 0.01, ***P < 0.001 compared with the sh-NC group; ^#^ *P* < 0.05, ^##^ *P* < 0.01 compared with the sh-AC125257.1-1/-2 group.

By CCK-8 assay, SW480 and HCT-8 transfected with sh-AC125257.1 showed suppressed viability, while those transfected with sh-AC125257.1-1/-2 + CASC5 partially reserved this effect (Figure 5a). As is shown in flow cytometry results, the apoptosis rate of SW480 and HCT-8 transfected with sh-AC125257.1 was remarkably increased, while group transfected with sh-AC125257.1-1/-2 + CASC5 showed reversed effect (Figure 5b). Further western blot analysis clarified the protein changes (Figure 5c). To be specific, CASC5, BCL2, and CDK4 levels were decreased by knockdown of AC125257.1, and BAX level was increased by it, suggesting that CRC cell apoptosis was inhibited by AC125257.1. The expression of Cyclin A1 and Cyclin D1 was downregulated, suggesting that the knockdown of AC125257.1 suppressed CRC progression. These effects could all be reversed partially by CASC5.

### 3.6 Knockdown of AC125257.1 suppressed CRC tumor growth *in vivo*

**Figure 6:**
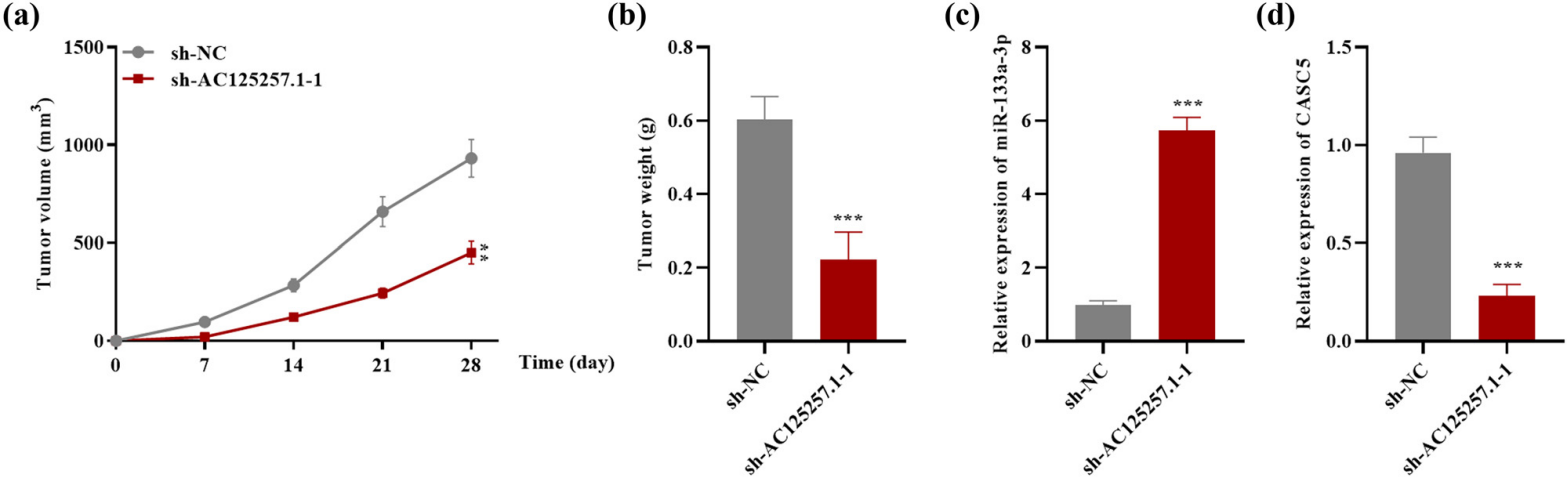
(a) Tumor volume in each group was measured on days 7, 14, 21, and 28. (b) Mouse tumor weight in the sh-NC and sh-AC125257.1-1 groups. (c) RT-qPCR was used to detect the expression of miR-133a-3p in mouse tumor tissues in each group. (d) The expression of CASC5 was measured using RT-qPCR mouse tumor tissues in each group. **P < 0.01, ***P < 0.001.

Xenograft tumor-bearing mouse models were established to examine the function of AC125257.1 on CRC tumorigenesis. The results indicated that AC125257.1 knockdown significantly decreased the tumor growth rate, and mouse tumor volume and weight was significantly smaller in the sh-AC125257.1-1 group compared with the control group (Figure 6a and b). Moreover, we examined the expression of miR-133a-3p and CASC5 in mouse tumor tissues. As revealed by RT-qPCR analysis, the expression of miR-133a-3p was significantly increased while the CASC5 levels showed a significant decrease in the sh-AC125257.1-1 group compared with the control group (Figure 6c and d).

## 4 Discussion

In the present study, we found that lncRNA AC125257.1 was upregulated in CRC tissues and cells, and AC125257.1 silencing inhibited the proliferation and promoted the apoptosis of CRC cells by binding with miR-133a-3p to upregulate CASC5. AC125257.1 silencing also showed suppressive effects on CRC tumor growth *in vivo*. These findings indicated the potential of AC125257.1 as a biomarker and therapeutic target for the treatment of CRC.

Previous study has shown that CASC5 can promote CRC malignancy through m6A methylation [23], suggesting that CASC5 plays an oncogenic role in CRC. Besides CASC5, other potential targets of miR-133a-3p, such as UBA2 and AQP1, are revealed to be implicated in CRC progression. For example, miR-133a has been reported to inhibit the CRC development and metastasis by regulating UBA2 [24]. miR-133a-3p suppresses CRC cell proliferation, migration, and invasion by targeting AQP1 [25]. In this study, we confirmed that CASC5 was abundant in CRC cells and tumor tissues with poor prognosis. Several reports illustrated that miR-133a-3p suppresses CRC development, which is consistent with our findings [25,26,27]. The inner interaction between miR-133a-3p and CASC5 was analyzed by the RNA pull-down assay. We found that CASC5 is targeted by miR-133a-3p. Meanwhile, CASC5 restored the inhibitory effect of miR-133a-3p in CRC cell lines.

As with the data from the TCGA database, we also found that AC125257.1 had high expression in CRC samples (cells and tumor tissues). High expression of AC125257.1 is associated with poor prognosis in CRC patients. lncRNA with multiple cellular functions are closely linked with human cancer progression [28] and has emerged to have the potential as therapeutic target for CRC [29]. The lncRNA-mediated ceRNA regulatory network is an important pathogenic mechanism of CRC [30]. Yu et al. claimed that CXCL12/CXCR4 sponging miR-133a-3p promoted CRC progression [31]. To explore the novel potential ceRNA regulatory network associated with miR-133a-3p, we verified that AC125257.1 can sponge miR-133a-3p by RIP and RNA pull-down experiments. Furthermore, the knockdown of AC125257.1 promoted miR-133a-3p expression and suppressed CASC5 expression, thereby inhibiting CRC cell viability, revealing that AC125257.1 regulates CRC progression by mediating the miR-133a-3p/CASC5 axis. Besides miR-133a-3p, other potential miRNAs binding to AC125257.1, such as miR-944 and miR-133b, are reported to affect CRC development. For example, miR-944 is revealed to suppress the proliferation, migration, and invasion of CRC cells by targeting GATA6 in CRC [32]. miR-133b is reported to reduce the stemness and chemoresistance in colorectal spheroids by regulating DOT1L [33]. The findings of our study may contribute to understanding the regulatory mechanism of CRC.

However, there remained limitations of our study. First, a further expansion of CRC sample size is needed to obtain a more general data. Second, starBase predicted the presence of other miRNA and genes targeted by AC125257.1 and miR-133a-3p, suggesting other pathogenic mechanisms of CRC.

Taken together, these results suggested that knockdown of AC125257.1 inhibits CRC development *in vitro* and *in vivo* via miR-133a-3p/CASC5 axis. Our study deepened our understanding of the pathogenesis of CRC. The study also suggested that high levels of CASC5 may be a marker affecting overall survival in CRC patients.
